# The Role of Polymer Chain Stiffness and Guest Nanoparticle Loading in Improving the Glass Transition Temperature of Polymer Nanocomposites

**DOI:** 10.3390/nano13131896

**Published:** 2023-06-21

**Authors:** Raja Azhar Ashraaf Khan, Mengbo Luo, Ahmad M. Alsaad, Issam A. Qattan, Sufian Abedrabbo, Daoyang Hua, Afsheen Zulfqar

**Affiliations:** 1 Department of Physics, Zhejiang Normal University, Jinhua 321004, China; azharashraaf@gmail.com (R.A.A.K.);; 2 Department of Physics, Zhejiang University, Hangzhou 310027, China; 3 Department of Physics, Jordan University of Science & Technology, P.O. Box 3030, Irbid 22110, Jordan; alsaad11@just.edu.jo; 4 Department of Physics, Khalifa University of Science and Technology, P.O. Box 127788, Abu Dhabi 127788, United Arab Emirates; sufian.abedrabbo@ku.ac.ae

**Keywords:** polymer, nanocomposites, stiffness, glass transition temperature, nanoparticles

## Abstract

The impact of polymer chain stiffness characterized by the bending modulus (*k_θ_*) on the glass transition temperature (*T*_g_) of pure polymer systems, as well as polymer nanocomposites (PNCs), is investigated using molecular dynamics simulations. At small *k_θ_* values, the pure polymer system and respective PNCs are in an amorphous state, whereas at large *k_θ_* values, both systems are in a semicrystalline state with a glass transition at low temperature. For the pure polymer system, *T*_g_ initially increases with *k_θ_* and does not change obviously at large *k_θ_*. However, the *T*_g_ of PNCs shows interesting behaviors with the increasing volume fraction of nanoparticles (*f*_NP_) at different *k_θ_* values. *T*_g_ tends to increase with *f*_NP_ at small *k_θ_*, whereas it becomes suppressed at large *k_θ_*.

## 1. Introduction

The glass transition temperature (*T*_g_) and melting temperature (*T_m_*) are two important properties of polymers related to the order of polymer chains in any polymer system [1]. The degree of ordering of chains in the bulk mainly depends on stiffness which can be adjusted by tuning the bending modulus (*k_θ_*) in the simulations incorporated via the bending potential given in Equation (3) [2,3,4]. A finitely long polymer chain behaves like a flexible polymer at smaller values of *k_θ_* and it turns into a rod-like structure at high enough values of *k_θ_*. Experimentally, the stiffness of a polymer chain can be tailored by introducing some functional groups or by initiating crosslinking [5]. During the annealing process of an amorphous polymer system (with no long-range order of polymer chains), the transition of a glass state into a rubber state takes place at a particular temperature referred to as *T*_g_ while *T_m_* is referred to as a phase transition of a crystalline polymer system (with a higher degree of polymer order) into its liquid form. The partial polymer crystalline order can be witnessed in long semiflexible neutral polymer systems while a higher degree of polymer order can be observed in highly stiff short polymers [6,7,8,9]. An increase in polymer concentration and stiffness can trigger a transition of a polymer system from a highly disordered (isotropic) to a relatively ordered (nematic) state [6,10,11,12,13]. The crystallinity of the polymer can be witnessed in several polymer nanocomposites (PNCs) along with several biological systems [14,15,16].

Natural and synthetic polymers exhibit a lot of variations in their properties depending on stiffness, especially *T*_g_ [17]. An understanding of *T*_g_ is of significant importance and has continued to spark intense discussion among scientists [18,19,20,21,22,23]. The glass transition is not only limited to conventional polymers but also extends to other important partially stiff biopolymers, such as DNA and proteins [24,25]. The polymer chain stiffness also influences the static and dynamic behaviours of the polymer [26,27]. It was pointed out that the diffusion of polymer strands can also influence various cellular functions [28]. The dynamics of biological polymers are dependent on the *T*_g_ and are also distorted by guest filler loading [29,30,31,32,33,34,35,36,37]. For example, fillers such as cytoskeleton, actin, nucleus, and additive chromatins, etc., significantly alter the dynamics of living polymers such as RNAs and proteins [30,34,38,39,40]. The arrangement of polymer chains in any system is also largely reliant on the interplay between chain rigidity and entropy [24,27,41,42,43].

The guest nanoparticles (NPs) can be incorporated into the host polymer system to enhance their macroscopic characteristics [44,45,46,47,48,49,50,51,52,53,54,55]. So, the polymer/NP systems have countless applications in technological fields along with industrial formulations [46,49,52]. The impact of the loading of NPs on a polymer’s dynamical and structural properties is a hot research topic [44,56,57,58,59,60,61,62,63,64]. The size and loading percentage of NPs have a substantial impact on the dynamics and conformational characteristics of polymers and hence the *T*_g_ [56,57,58,59,60,61,65]. Moreover, it was also revealed that the attraction between polymers and NPs significantly alters the properties of polymers [46,57,58,66]. For example, experiments on PMMA nanocomposites with silica NPs reported a significant improvement in the *T*_g_ due to the strong attraction generated by the substantial surface charge density of NPs towards PMMA [58]. An improvement in the *T*_g_ of polyimide (PI) nanocomposite with attractive octa(aminophenyl) polyhedral oligomeric silsesquioxane (OAPS) NPs was also reported, whereas the change in *T*_g_ was almost negligible with repulsive octaphenyl silsesquioxane (OPS) NPs [66]. Atomistic molecular dynamics simulations reported a significant influence on the dynamics of the polymer interface with silica NP [67]. The change in the stiffness of a polymer chain can also influence the *T*_g_ of polymer bulk and PNCs. For example, coarse-grained molecular-dynamics simulations demonstrated that the *T*_g_ of polymer bulk with three types of chain stiffness, i.e., freely jointed chain (FJC), freely rotating chain (FRC), and rotation isomeric state (RIS) with a stiffness order of FJC < FRC < RIS, increases by increasing polymer chain stiffness [68]. However, this aspect in the case of PNCs has rarely been studied so far. For instance, one such study that has some partial relevance with polymer chain stiffness and the *T*_g_ of PNCs was carried out by Podsiadlo et al. [1] in early 2000. Their differential scanning calorimetry (DSC) study on PNCs of PVA and montmorillonite (MTM) crosslinked with glutaraldehyde (GA) showed significant improvement in the *T*_g_ because of the improvement in PVA stiffness. However, for a better understanding of such systems, a deep molecular dynamics simulation study is still required.

In this paper, we investigate the impact of the stiffness of polymer chain and volume fraction of NPs on the *T*_g_ of pure polymer and PNCs excluding the melting temperature because the underlying physics behind both phenomena is the same for any possible shift. We find that NPs interact differently with polymer chain stiffness in PNCs and, hence, the *T*_g_ shows different trends with the increasing volume fraction of NPs.

Polymers are thermally less stable and exhibit a lot of variation in their stiffness depending on the type of polymer. So, the nanoparticles are added to improve the stability of polymers. To date, it is not clear whether the loading of NPs increases the thermal stability of polymers or in some cases can suppress it. In this work, we considered polymer chains with different chain stiffnesses and loaded attractive NPs to explore the impact on *T*_g_. We expect that this study could be helpful in understanding the *T*_g_ of various PNC systems consisting of polymer chains with a wide range of stiffnesses.

## 2. Model and Simulation Method

The *T*_g_ and other properties of pure polymer and PNCs with mobile and attractive NPs are investigated in a crowded environment using molecular-dynamics simulations. The system consists of a total number of *n* = 96 chains with the same polymer length *N* = 44. A large system size (greater than 5<*R*_g_> with <*R*_g_> as the mean radius of gyration of polymer in bulk) is adopted here to minimize the size effect. The volume fraction of NPs, *f*_NP_, is defined as:

(1) $$f_{NP}=\frac{\pi }{6}\frac{{N_{NP}\sigma }_{NP}^{3}}{V}$$

**Figure 1:**
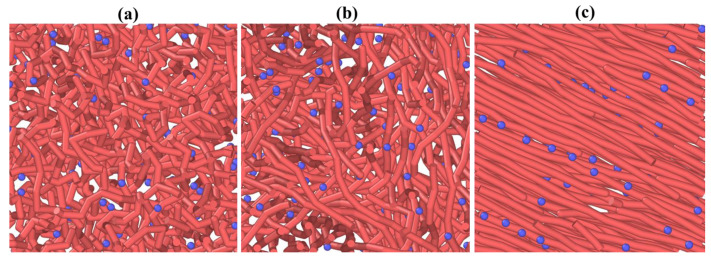
A 2D representation of systems consisting of polymer chains and NPs (**a**) *k_θ_* = 1, (**b**) *k_θ_* = 10, and (**c**) *k_θ_* = 40 at *f*_NP_ = 0.06. Blue beads are NPs while worm-like radish structures represent the polymer chains.

Here, the diameter of NPs is represented by *σ*_NP_ and the quantity of NPs in the system by *N*_NP_. The simulations are conducted in a cubic simulation system with a volume *V* = *L*^3^, where *L* is the length of a cubical box. Along the *x*, *y*, and *z* directions, periodic boundary conditions (PBCs) are used. We have investigated the *T*_g_ in two ways: at different values of *k_θ_* and at different values of *f*_NP_. Various samples were introduced, i.e., from *k_θ_* = 1 representing a flexible polymer chain to *k_θ_* = 60 representing a highly ordered semicrystalline polymer chain. Since the polymer chains’ order at *k_θ_* = 60 is relatively high, we restricted ourselves up to *k_θ_* = 40 only for PNC cases. The volume fraction of NPs is tailored from *f*_NP_ = 0 to *f*_NP_ = 0.15. Figure 1a–c present different systems with *k_θ_* = 1, *k_θ_* = 10 and *k_θ_* = 40, respectively, at *f*_NP_ = 0.06. An increase in the polymer chains’ order with increasing polymer chain stiffness is obvious. In our simulation system *f*_NP_, polymer chain stiffness and the NP–polymer interaction strength can be regulated; however, we preferred to work with a constant NP–polymer interaction strength. The *T*_g_ was studied by monitoring the temperature–volume relations of the system. The diffusivity of polymer chains and conformational properties were calculated by running several NPT simulations.

The linear polymer chains employed in the simulation are made up of *N* identical, spherical monomers that have size *σ,* equal to the size of a NP, i.e., *σ* = *σ_NP_* = 1. In various simulation systems, mainly four types of interactions are involved. Each interaction has a distinct potential. The interaction between bonded monomers in a chain is represented by the FENE potential given by Equation (2). To control polymer chain stiffness, Equation (3) is used. For the interactions between non-bonded monomers, between monomers and NPs, and between NPs, Equation (4) is used [69,70,71].

(2) $$V_{\mathrm{FENE}}(b)=\left\{\begin{array}{ll} -\frac{1}{2}KR_{0}{}^{2}\ln\left[1-{\left(\frac{b}{R_{0}}\right)}^{2}\right]+4\varepsilon_{\mathrm{PP}}\left[{\left(\frac{\sigma }{b}\right)}^{12}-{\left(\frac{\sigma }{b}\right)}^{6}+\frac{1}{4}\right] & ,\;b<R_{0}\\ \infty & ,\;b\geq R_{0} \end{array}\right.$$

(3) $$V_{\mathrm{bend}}(\theta )=k_{\theta }(1+\cos\theta )$$

(4) $$V_{\mathrm{ij}}^{\mathrm{LJ}}(r)=\left\{\begin{array}{ll} 4\varepsilon_{\mathrm{ij}}\left[{\left(\frac{\sigma }{r}\right)}^{12}-{\left(\frac{\sigma }{r}\right)}^{6}\right]-4\varepsilon_{\mathrm{ij}}\left[{\left(\frac{\sigma }{r_{c}}\right)}^{12}-{\left(\frac{\sigma }{r_{c}}\right)}^{6}\right] & ,\;r<r_{c}\\ \;0 & ,\;r\geq r_{c} \end{array}\right.$$

In Equation (2), *b* represents the bond length, *K* is the elastic coefficient and *R*_0_ is the longest bond length that each bond may extend. We set *K* = 30*ε*_PP_/*σ*^2^ and *R*_0_ *=* 1.5*σ* in this work. In Equation (3), *V*_bend_(*θ*) represents the bending potential with *k_θ_* the bending modulus and *θ* the bond angle between two successive bonds in a polymer chain [3]. In Equation (4), indices *i* and *j* correspond to the different species (P for polymer monomer and N for NP). The interaction strengths are denoted as *ε*_PP_, *ε*_NP_, and *ε*_NN_, respectively. The cutoff distances are set as *r_c_ =* 2.5*σ* for monomer–monomer and monomer–NP interactions and *r*_c_ = 2^1/6^*σ* for NP–NP interactions. Here, the cutoff at *r*_c_ = 2^1/6^*σ* enables pure repulsion between NPs.

The Langevin dynamics equation describes how polymer monomers and NPs move across the simulation system.

(5) $$m_{i}\frac{{d^{2}r}_{i}}{dt^{2}}=F_{c}-m_{i}\Gamma_{i}v_{i}+F_{r}$$

Here, *F_c_* is the conservative force in the system, i.e., $F_{c}=-\nabla_{i}\sum \left(V_{\mathrm{FENE}}+V_{\mathrm{PP}}^{\mathrm{LJ}}+V_{\mathrm{PN}}^{\mathrm{LJ}}+V_{\mathrm{NN}}^{\mathrm{LJ}}\right)$ and the summation is applied to all NPs and monomers. The viscous damping force is expressed by the second term, where *Γ_i_* is the frictional coefficient. For polymer monomers and NPs, *Γ_i_* is configured to be a constant. The final term takes into account the white noise force, which has a zero mean and a correlator $<F_{r}(t_{1})\cdot F_{r}(t_{2})>=6m_{i}\Gamma_{i}k_{B}T\delta (t_{1}-t_{2})$. Here, $\delta (t_{1}-t_{2})$ denotes the Dirac delta-function. There are no correlations between the thermal noise force and multiple cartesian directions. The mass of NP, *m*_NP_, is determined by the size and density of the NP. In the current simulations, we used *m*_NP_ = 1.

LJ units are used to express each physical quantity. We set *m =* 1, *σ =* 1 and *ε*_PP_ *=* 1 as the corresponding units for mass, length, and energy, respectively. The reduced unit for time is $\tau_{0}=\sqrt{\frac{m\sigma^{2}}{\varepsilon_{PP}}}$. The unit of temperature is *ε*_PP_/*k*_B_ with *k*_B_, the Boltzmann’s constant. We fix *ε*_NP_ = 2 and *ε*_NN_ = 1 in this work. Polymers and NPs can be mixed very well at large ε_NP_.

The *T*_g_ and the other characteristics of pure polymer and PNC systems are simulated by using Large-scale Atomic/Molecular Massively Parallel Simulator (LAMMPS) software (https://www.lammps.org/download.html, accessed on 15 April 2023) [72]. LAMMPS software has been extensively used for PNC systems [73,74]. We adopted NPT simulations using a Langevin thermostat with a damping constant *Γ* = τ_0_^−1^ and a damping parameter for pressure 0.001τ_0_^−1^. The pressure *P* = 1 is set as constant during the whole simulation. The time step is Δ*t* = 0.0001τ_0_. In the current study, we calculated the *T*_g_ using the system’s volume–temperature relation at constant *P*. Firstly, we created a low density (*ρ =* 0.5) simulation box. Secondly, we slowly compressed the whole system along all the sides at a steady rate to produce the required pressure at *T* = 3. In the third step, the equilibrium of the system was achieved at *T* = 3 after a long simulation run (about 3 × 10^7^τ_0_). The system was assumed to be in equilibrium when the fluctuation of volume versus time was small. Ultimately, we conducted a simulated annealing process from a higher temperature *T* = 3 to a lower temperature 0.02 by using an NPT ensemble at *P* = 1 [75]. The annealing time consumed during this process is about 5.5 × 10^7^τ_0_. However, the production run was only considered from *T* = 2.5 to 0.02, neglecting the melting phase at higher temperatures. A slope change spot was detected from the volume versus temperature plot to determine the *T*_g_. To study the diffusion and other properties of the polymer, we performed another equilibrium step at *T* = 2, not far from the *T*_g_ for a sufficiently long time (20 × 10^7^τ_0_). The simulation results are averaged over 2–5 independent samples to make sure that the difference in the result is no more than 6% for various samples.

## 3. Results and Discussion

With the increase in the stiffness of a polymer chain, it is possible that a transformation of a polymer chain system from an amorphous state to a semicrystalline or crystalline state takes place. In the crystalline state, polymer chains behave like a highly ordered structure. To visualize the transformation of an amorphous state to a highly ordered semicrystalline state (when *k_θ_* is increased), we first calculate the nematic order parameter ($S_{2}$) as follows:

(6) $$S_{2}=\frac{1}{2}<3{\left|\hat{u}\cdot \hat{n}\right|}^{2}-1>$$

Here, *S*_2_ and the director ($\hat{n}$) can be calculated by solving the tensor matrix:

(7) $$Q=\frac{1}{n}\sum_{i=1}^{n}\left(\frac{3}{2}{\hat{u}}_{i}⊗{\hat{u}}_{i}-\frac{1}{2}I\right)$$

with *I*, the unit second-rank tensor. Here *S*_2_ is the largest eigenvalue and $\hat{n}$ the relevant unit eigenvector of the matrix *Q* [76,77,78].

**Figure 2:**
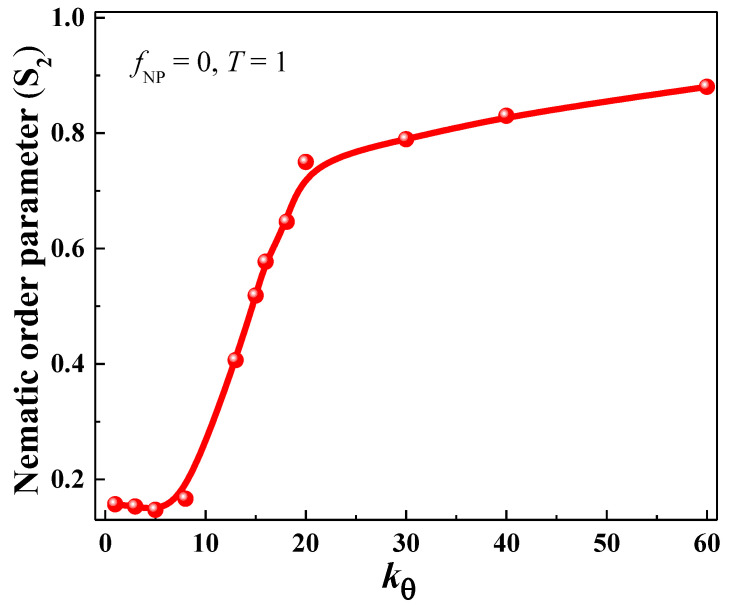
The nematic order parameter ($S_{2}$) versus *k_θ_* plot at *f*_NP_ = 0. The transition from the amorphous state to a highly ordered semicrystalline state is obvious.

The evolution of the nematic order parameter ($S_{2}$) with *k_θ_* at *f*_NP_ = 0 is shown in Figure 2. It is clear that $S_{2}$ is strongly dependent on *k_θ_*. We can divide the $S_{2}$ vs. *k_θ_* curve behavior into three distinct parts: part I: *k_θ_* = (1–10), part II: *k_θ_* = (10–20) and part III: *k_θ_* = (20–60). In part I, $S_{2}$ does not change significantly and remains constant throughout this interval with an average value of $S_{2}$ at around 0.16. However, in part II, $S_{2}$ increases sharply with *k_θ_* as indicated by the steep slope in this particular interval which means a transformation of the polymer chain from an amorphous to a semicrystalline state takes place at a faster rate. From *k_θ_* = 20 onwards_,_ $S_{2}$ continues to increase at a slower rate (smaller slope than that in part II) until it reaches a maximum value of 0.88 at *k_θ_* = 60, indicating more order in the semicrystalline state. The variation of $S_{2}$ with polymer chain stiffness observed here is in agreement with similar works presented in the literature [2,77].

### 3.1. Glass Transition and Diffusion in Bulk Polymer System

**Figure 3:**
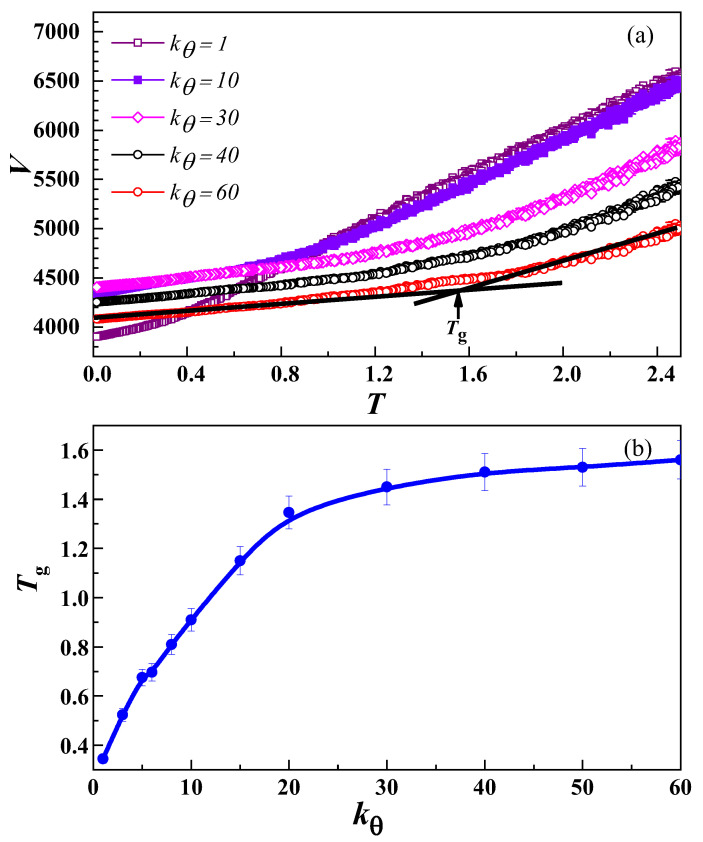
(**a**) The plot of volume of the system *V* against temperature *T* for different values of chain stiffness *k_θ_* at *f*_NP_ = 0. (**b**) The corresponding variation of the glass transition temperature *T*_g_ versus *k_θ_*.

Figure 3a presents the variation of *V* with *T* at different values of *k_θ_* for various systems containing only polymer chains. For every *k_θ_*, the total number of polymer chains, *n,* is equal to 96, where each chain length, *N*, is equal to 44. The *T*_g_ is defined by the *V*–*T* curve, i.e., the temperature at which the behaviour of the slope of the curve changes differently. Figure 3a shows that *T*_g_ depends on *k_θ_*, which is varied from *k_θ_* = 1 to *k_θ_* = 60 in the current study. At small *k_θ_* values, the change in system volume is larger than that at high *k_θ_*. This difference is mainly due to the increase in semicrystalline regions with increasing *k_θ_* values.

The values of *T*_g_ at different *k_θ_* are also displayed in Figure 3b. Initially, with the increase in *k_θ_*, more amorphous regions turn into ordered states; so, an increasing behaviour of the *T*_g_ is noticed, i.e., *T*_g_ increases from the lowest value of 0.3 at *k_θ_* = 1 up to a higher value of 1.45 at *k_θ_ =* 20. After that (*k_θ_* > 20), the increase in *T*_g_ slows down notably until it reaches a maximum value and levels off, indicating that more crystalline regions are already formed.

**Figure 4:**
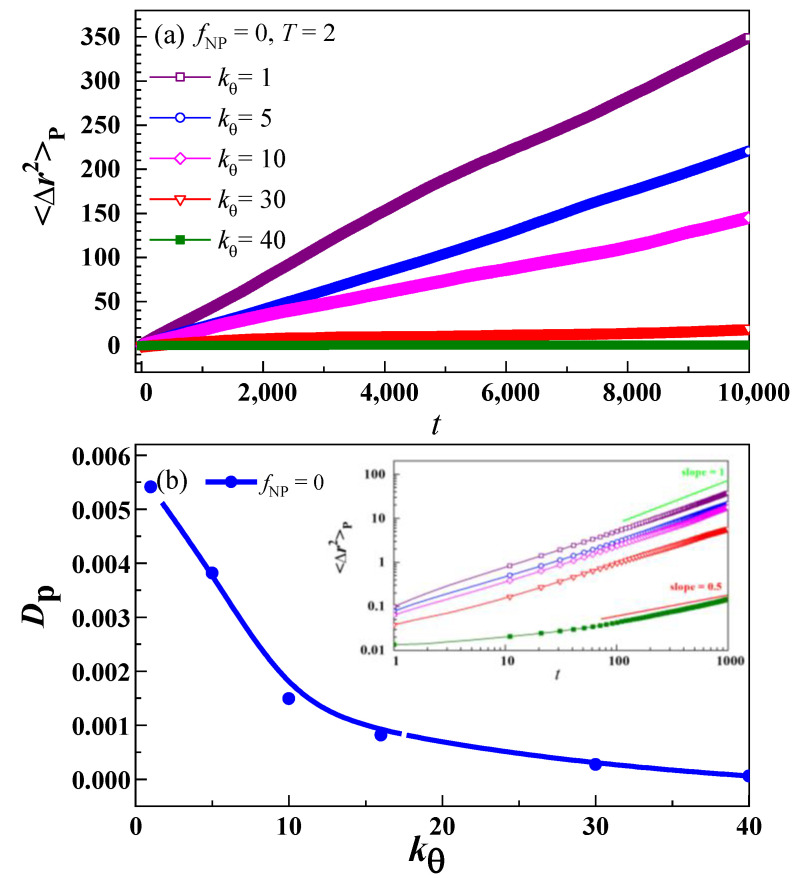
(**a**) Plot of the mean square displacement of the polymer <Δ*r*^2^>_P_ as a function of time at different values of *k_θ_* at *f*_NP_ = 0 and *T* = 2. (**b**) the overall dependence of diffusion constant *D*_P_ on *k_θ_*. The inset presents the slope varying from 0.5 (Rouse model prediction) to 1 (Einstein diffusion).

To verify the *T_g_* behaviour with *k_θ_* depicted in Figure 3b, we looked into the diffusivity of the polymer at *T* = 2, which was higher than *T*_g_. The diffusion of Polymer (*D*_P_ = <Δ*r*^2^(*t*)>_P_/6*t*) is calculated from the MSD equation $\langle \Delta r^{2}\left(t\right)\rangle =\langle {\left[r_{cm}\left(t\right)-r_{cm}\left(0\right)\right]}^{2}\rangle$. The changes in MSD <Δ*r*^2^(*t*)>_P_ in relation to *f*_NP_ = 0 at different values of *k_θ_* are shown in Figure 4a. The MSD <Δ*r*^2^(*t*)>_P_ of the polymer increases with time. Although at high stiffness (where the nematic order is high) the chain translation becomes highly anisotropic, but since we are interested in the overall behavior of the pure polymer and PNC system, we will not discuss anisotropic characteristics here [67].

Figure 4b describes the diffusion constant *D*_P_ behaviour at different *k_θ_* values. The variation of *D*_P_ and *T*_g_ are consistent with one another, i.e., as *T*_g_ increases at a faster rate, *D*_P_ decreases at a similar rate and vice versa for the same *k_θ_* values. These results indicate that the calculated *T*_g_ values of polymer systems are strongly inversely related to the diffusion of polymer. The inset of Figure 4b shows the log–log plot of MSD with time. It is interesting to see that, at lower values of *k_θ_*, the slope of MSD curves is around 1 (Einstein diffusion), and at higher values, especially at *k_θ_* = 40, the slope is around 0.5 (the prediction of the Rouse model). So, the final normal diffusion of polymer chains is not achieved for large stiffnesses as our simulation time is not long enough [79,80]. It is also interesting to see how the guest NPs interact with the polymer (having different stiffness) and shift the *T*_g_, as no detailed study is available so far regarding this phenomenon. So, we have also studied the effect of NPs on the *T*_g_ of PNCs consisting of polymers with variable stiffness.

### 3.2. Glass Transition in Polymer Nanocomposites

**Figure 5:**
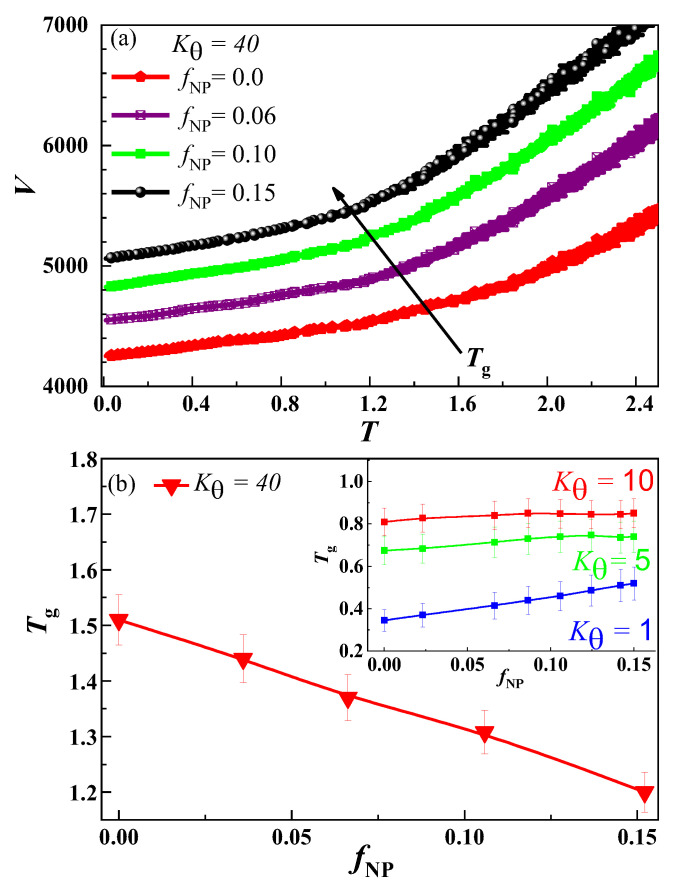
(**a**) At *k_θ_* = 40, system volume *V* variations with temperature *T* for different *f*_NP_. (**b**) The variation of *T*_g_ with *f*_NP_ at *k_θ_* = 40. The inset shows the variation of *T*_g_ with *f*_NP_ at *k_θ_* = 1, 5 and 10, respectively.

Figure 5a shows the change in system volume *V* with respect to *T* at different values of *f*_NP_ for *k_θ_* = 40. It is obvious that the *T*_g_ is heavily dependent on *f*_NP_. The values of *T*_g_ obtained from *V*–*T* curves at different *f*_NP_ for *k_θ_* = (1, 40) are plotted in Figure 5b.

The volume fraction of NPs is varied from *f*_NP_ = 0 to *f*_NP_ = 0.15 while keeping other parameters (*N, n*) intact. A linear decreasing behaviour of *T*_g_ is noticed for the *k_θ_* = 40 case, which is totally different from *T*_g_ behaviour for other *k_θ_* values shown in the inset of Figure 5b. Results show a fast increase in *T*_g_ at *k_θ_* = 1 that is consistent with our recent work [81], a slight increase in *T*_g_ at *k_θ_* = 5 and a negligible effect of *f*_NP_ on *T*_g_ at *k_θ_* = 10. Recently, we showed that the increase in *T*_g_ at *k_θ_* = 0 is due to the fraction of monomers contacted with NPs at low *f*_NP_. So, here it will be interesting to calculate the fraction of monomers contacted with the NPs (*f*_contact_) at different *k_θ_* and *f*_NP_. Generally, the extent of *f*_contact_ is mainly dependent on the type of interaction between polymer monomers and NPs, its strength and the volume fraction of NPs.

**Figure 6:**
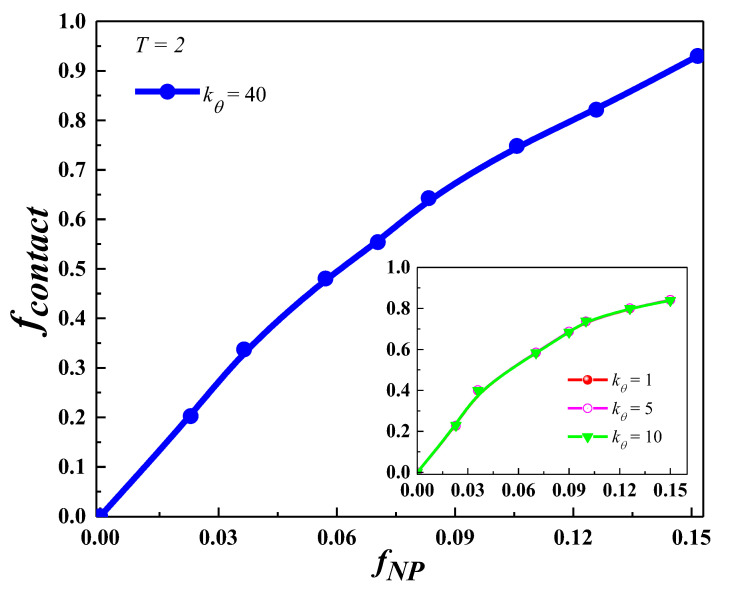
Plot of the fraction of monomers contacted with nanoparticles *f*_contact_ with *f*_NP_ at *k_θ_* = 40. The inset shows the same with *k_θ_* = 1, 5 and 10 at *T* = 2.

Figure 6 shows the variation of *f*_contact_ with *f*_NP_ at *k_θ_* = 1, 5, 10, and 40. Here, a monomer is considered as contacted with a NP if the monomer–NP center-to-center distance is less than 0.5*σ*_NP_ + *σ* = 1.5*σ.* We noticed that at *k_θ_* = 1, 5 and 10, *f*_contact_ is almost independent of polymer chain stiffness. However, at *k_θ_* = 40, *f*_contact_ is slightly higher than at other *k_θ_* values.

**Figure 7:**
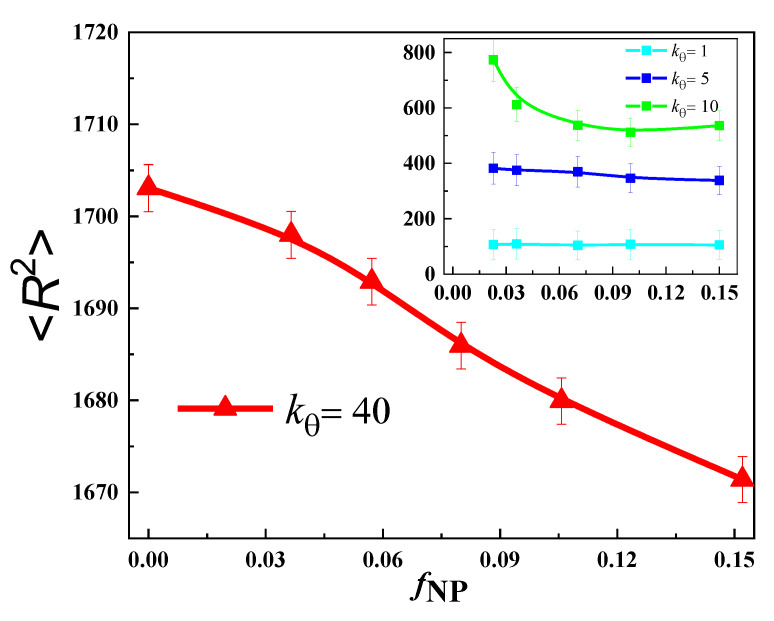
The variation of mean square end-to-end distance (<*R*^2^>) with *f*_NP_ at *k_θ_* = 40. The inset shows the dependence of <*R*^2^> with *f*_NP_ at other values of *k_θ_*, i.e., *k_θ_* = 1, 5 and 10 at *T* = 2.

We have also studied the impact of nanoparticle loading on the mean square end-to-end distance (<*R*^2^>) of the polymer at different *k_θ_*, as shown in Figure 7. It is noticed that at *k_θ_* = 1, there is a small change of about 4% in <*R*^2^> when *f*_NP_ is increased to a maximum value of 0.15. Similarly, at *k_θ_* = 5, we see a change of about 12% in <*R*^2^>, at *k_θ_* = 10 about 30%, and at *k_θ_* = 40 just a change of 1.8%. So, we can conclude that the size of very flexible and very stiff polymers is least affected by the presence of nanofillers.

**Figure 8:**
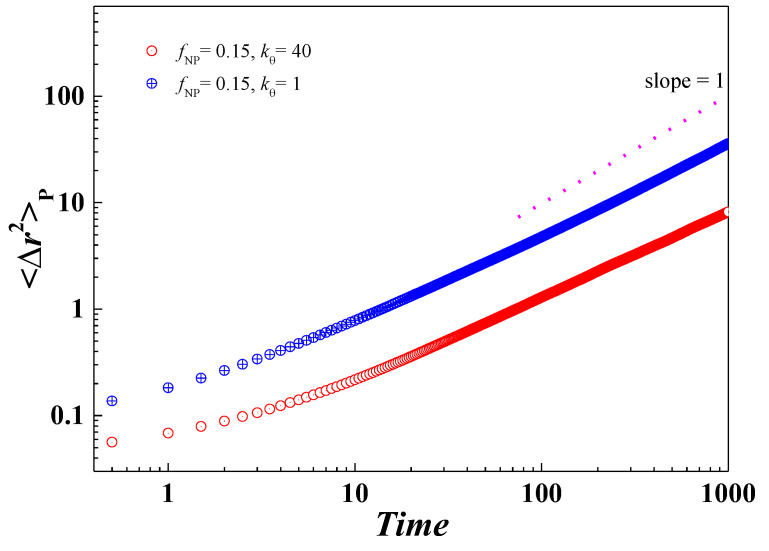
The log–log plot of the mean square displacement of the polymer in nanocomposite (*f*_NP_ = 0.15) as a function of time at *k_θ_* = 1 and *k_θ_* = 40.

Finally, it is also important to check if there is any change in the diffusion mode of polymers in PNC. For this purpose, we chose a constant volume fraction of NPs (*f*_NP_ = 0.15) and different values of polymer chain stiffness, i.e., *k_θ_* = 1 and *k_θ_* = 40, as shown in Figure 8. It is interesting to see that at *k_θ_* = 1, the polymer undergoes diffusion close to the normal Einstein diffusion as is also presented for a pure polymer system in Figure 4b. However, we observed a significant change in the case of *k_θ_* = 40. For the pure polymer, we revealed diffusion as predicted by the Rouse model, but in the case of nanocomposites, a transformation towards normal Einstein diffusion was observed that is consistent with the *T*_g_ behavior.

## 4. Conclusions

The glass transition temperature of pure polymer and respective nanocomposites consisting of different polymer chain stiffnesses (*k_θ_*) and loadings of nanoparticles is investigated in the current study using MD simulation. We revealed that at small *k_θ_*, pure polymer systems and polymer nanocomposites (PNCs) exhibit a glass transition at low temperature. For a pure polymer system, *T*_g_ initially increases with the increase in *k_θ_*, and it does not change obviously at higher values of *k_θ_*. However, in the case of PNC, *T*_g_ changes differently with the increasing volume fraction of NPs (*f*_NP_) at different *k_θ_* values. As the *f*_NP_ is increased, *T*_g_ increases rapidly at *k_θ_* = 1, slowly at *k_θ_* = 5, and changes negligibly at *k_θ_* = 10. At large enough *k_θ_*, e.g., *k_θ_* = 40, it is noticed that *T*_g_ decreases with increasing *f*_NP_. We have also demonstrated that in the case of polymer nanocomposite, the fraction of monomers contacting with NPs (*f*_contact_) is not affected by the changing polymer chain stiffness. However, NPs obviously affect the mean square end-to-end distance (*<R*^2^*>*) of the polymer at different stiffnesses of polymer chains. Due to the unique behaviors of the PNC systems explored here, our study would be helpful in the development of more stable polymer nanocomposites in the future.

## Data Availability

Data available on request.
